# Metabolomic Screening of Anti-Inflammatory Compounds from the Leaves of *Actinidia arguta* (Hardy Kiwi)

**DOI:** 10.3390/foods8020047

**Published:** 2019-02-01

**Authors:** Gyoung-Deuck Kim, Jin Young Lee, Joong-Hyuck Auh

**Affiliations:** Department of Food Science and Technology, Chung-Ang University, Anseong 17546, Korea; kberonika@gmail.com (G.-D.K.); j_young_2@naver.com (J.Y.L.)

**Keywords:** Anti-inflammatory, *Actinidia arguta*, caffeic acid derivatives, metabolomic screening

## Abstract

The metabolomic screening of potential anti-inflammatory compounds in the leaves of *Actinidia arguta* was performed by using LC-MS/MS. Ethanol extracts were prepared, and the anti-inflammatory effects were investigated based on nitric oxide (NO) synthesis and inducible nitric oxide synthase expression in lipopolysaccharide-induced RAW 264.7 macrophages. The 75% ethanol extract showed the highest inhibitory effect on nitric oxide (NO) production, and it was further separated by in vitro bioassay-guided fractionation using preparative LC with reversed-phase column separation. Through multiple steps of fractionation, sub-fraction 1-3 was finally purified, and caffeic acid derivatives, such as caffeoylthreonic acid and danshensu (salvianic acid A), were successfully identified as key anti-inflammatory compounds by LC-MS/MS and metabolomics analyses. This is the first study identifying anti-inflammatory compounds in *A. arguta* (*Actinidia arguta*) leaves through bioassay-guided fractionation and metabolomics screening. Results of this study would be useful for the application of *A. arguta* leaves as a nutraceutical.

## 1. Introduction

*Actinidia arguta* (Actinidiaceae) is native to China, Korea, Siberia, and Japan. In Korea, fruits, stems, roots, and leaves of *A. arguta* (*Actinidia arguta*) are traditionally used as medicines. *A. arguta* exerts antioxidant, anti-inflammatory, and anti-proliferative activities [1,2,3]. Among the various parts, the leaves are used as a folk medicine to cure intestinal catarrh, stomach cancer, and acute gastritis in Korea [4]. Phytochemicals are extensively verified to provide health benefits, such as substrates for biochemical reactions, cofactors or inhibitors of enzymatic reactions, scavengers of reactive or toxic chemicals, and so on [5], and their compositions are significantly affected by agronomic and environmental conditions [6]. Phytochemicals in *A. arguta* leaves, such as flavonoids [7], lignin, and phenolic acid [8], have been reported as key anti-diabetic compounds. Webby et al. investigated flavonoids in the leaf of *Actinidia*, but the study was only focused on several compounds, such as quercetin, kaempferol, and myricetin, with their glycosides [7]. Recently, Kwak and Lee [9] also reported on the antioxidant and anti-inflammatory effects of ethanol extracts from *A. arguta* leaves, however, the systematic analysis of its key compounds has not been conducted.

Inflammation is one of the most important immune reactions protecting the body from harmful stimulus; however, prolonged and excessive inflammation induces many diseases, such as arthritis, osteoarthritis, diabetes, obesity, allergies, asthma, chronic bronchitis, cancer, and chronic gastritis. Controlling inflammation is of major importance in the treatment of illnesses associated with chronic inflammation. The role of nitric oxide (NO) in host defense and immune responses has been investigated, with an emphasis on inflammation responses. High levels of NO are produced in response to inflammatory stimuli, which then mediate pro-inflammatory cytokine release, tissue dysfunction, and organ damage [10,11]. NO is synthesized from L-arginine by a family of nitric oxide synthase (NOS) enzymes [10]. Three different isoforms of NOS have been characterized, such as neuronal NOS (NOS I), endothelial NOS (NOS III), and inducible NOS (iNOS, NOS II). Both neuronal NOS and endothelial NOS are constitutively expressed, and are inactive in resting cells. However, iNOS expression is not found in most resting cells. In addition, exposure to lipopolysaccharide (LPS) and/or pro-inflammatory cytokines induces the expression of iNOS in various inflammatory and tissue cells [10]. Thus, the use of selective iNOS inhibitors may be beneficial in the management of chronic inflammation [12]. Non-steroidal anti-inflammatory drugs are mostly used for the treatment of inflammatory diseases, despite their renal and gastric adverse effects [13], and medicinal plants are one of the useful sources of novel anti-inflammatory drugs and nutraceuticals [11].

Metabolomics is an emerging tool for the non-targeted profiling and identification of all metabolites in a sample under a given set of conditions [14,15]. Metabolomic data are processed by multivariate analyses [16]. Multivariate statistical analysis, such as principal component analysis (PCA) and orthogonal partial least squared-discriminant analysis (OPLS-DA), can clearly separate a data set into different groups, finally screening candidate metabolite for variation. This investigational approach facilitates the identification and profiling of the chemical characteristics of plants; it is also used in food science as a useful tool for analyzing bioactive compounds. In our previous study, pinoresinol–diglucoside was screened as a potential anti-diabetic compound in *A. arguta* leaves through metabolomic analysis [8]. In addition, ellagic acid in strawberry was identified as a key anti-inflammatory metabolite [17], and cyanidins in black raspberry were successfully screened as key bioactive substances countering adverse inflammation in murine macrophages [18]. These results indicate that a metabolomics approach is an appropriate method to identify bioactive compounds in functional foods. In this study, the anti-inflammatory effect of *A. arguta* leaves extract was evaluated in vitro, and active compounds were screened by partial purification through reversed-phase preparative LC (prep-LC). Key compounds were tentatively identified by LC-ESI-ion trap-MS/MS using multivariate statistical analysis.

## 2. Materials and Methods

### 2.1. Materials

Dulbecco’s modified Eagle’s medium (DMEM), HEPES, Dulbecco’s phosphate buffered saline (DPBS), penicillin-streptomycin, and fetal bovine serum (FBS) were obtained from Gibco BRL (Grand Island, NY, USA). Primary and secondary antibodies were purchased from Santa Cruz Biotechnology (Santa Cruz, CA, USA). HPLC grade solvents (methanol, ethanol, and acetonitrile) were purchased from Burdick & Jackson (Muskegon, MI, USA). LPS, 3-(4,5-dimethylthiazol-2-yl)-2,5-diphenyltetrazolium bromide (MTT), and formic acid were purchased from Sigma-Aldrich (St. Louis, MO, USA).

### 2.2. Preparation of Extracts

*A. arguta* leaves cultivated in Yangyang (a northeast region of South Korea) were collected and dried after blanching. Further, they were washed, air-dried, and homogenized (MCH-600, Tongyang, Seoul, Korea). Homogenized *A. arguta* leaves were extracted with water or different concentrations (25%, 50%, 75%, and 100%) of ethanol. The extracts were filtered (Whatman No. 1 filter paper, GE Healthcare, Buckinghamshire, UK) and concentrated by a rotary vacuum evaporator (Eyela, Tokyo, Japan) at 40 °C. Concentrated extracts were lyophilized to a dried powder and stored at −20 °C until use.

### 2.3. Partial Purification of the Extract

The 75% ethanol extract of *A. arguta* leaves was partially purified using preparative LC (LC-Forte/R, YMC Co., Kyoto, Japan). Separation was carried out using different gradients of acetonitrile or methanol with reversed-phase LC (YMC-DispoPack AT ODS, YMC-Triart Prep C18-S, YMC Co., Kyoto, Japan). UV spectra were measured at 270, 320, and 400 nm. The 75% ethanol extracts were separated into fractions 1–4 by YMC-DispoPack AT ODS. The flow rate was 25 mL min^−1^ with the solvents composed of 0.1% formic acid in water (A) and 0.1% formic acid in acetonitrile (B). The linear gradient profile was as follows: 0–50% of solvent B over 20 min, followed by 50–100% of solvent B over 10 min; the run was concluded at 30 min. Fraction 3, as an active fraction, eluted at 8–11 min, was further separated into sub-fractions 1–4 by YMC-Triart Prep C18-S (15 × 250 mm). The flow rate was 10 mL min^−1^ with the solvents composed of 0.1% formic acid in water (A) and 0.1% formic acid in methanol (B). The linear gradient profile was as follows: 10–30% of solvent B over 30 min, followed by 30–100% of solvent B over 10 min; the run was concluded at 40 min. The active fraction (sub-fraction 1 eluted at 11–21 min) was collected and further purified by YMC-Triart Prep C18-S (15 × 250 mm). The flow rate was 10 mL min^−1^ with the solvents composed of 0.1% formic acid in water (A) and 0.1% formic acid in methanol (B). The linear gradient profile was as follows: 10–20% of solvent B over 30 min; the run was concluded at 30 min. Finally, sub-fraction 1-3 (eluted at 17–19 min) was prepared as a partially purified active fraction of *A. arguta* extract and analyzed. Each fraction was collected according to the peak intensity chased at 270, 320, and 400 nm in the preparative LC profiles for the partial purification.

### 2.4. Cell Culture

RAW 264.7 cells were obtained from American type culture collection (ATCC, Rockville, MD, USA) and cultured in DMEM supplemented with 10% FBS, 1% penicillin-streptomycin, and 1% HEPES at 37 °C, and 5% CO_2_. For subculture, RAW 264.7 cells were rinsed with DPBS, mechanically scraped, and plated in 10-cm culture dishes.

### 2.5. Cell Proliferation Assay (MTT Assay)

Cell proliferation assay was performed by the MTT assay. RAW 264.7 cells were plated in a 96-well plate (4 × 10^4^ cells per well) and incubated overnight. Plated cells were treated with 0–100 μg/mL of samples and incubated for 20 h. MTT (5 mg/mL, 20 μL) was added to the cell suspensions and incubated for 4 h. After aspirating the medium from the wells, 200 μL of DMSO was added to dissolve the formazan crystals. The absorbance was measured using a microplate reader (Molecular devices, Sunnyvale, CA, USA) at 570 nm. Cell proliferation was described as the relative cell viability compared to that of control cells (medium alone, without sample treatment).

### 2.6. Measurement of Anti-Inflammatory Activity (NO Assay)

RAW 264.7 cells were plated in a 96-well plate (4 × 10^4^ cells per well) and incubated overnight at 37 °C and 5% CO_2_. Plated cells were treated with 100 ng/mL of LPS and samples for 20 h, and NO production from RAW 264.7 cells was quantified. After 20 h, the supernatants (50 μL) were mixed with 50 μL of sulfanilamide solution (1% sulfanilamide in 5% phosphoric acid) for 10 min at room temperature, and 50 μL of 0.1% *N*-1-napthylethylenediamine dihydrochloride in water was mixed for 10 min at room temperature. The absorbance at 540 nm was measured using a microplate reader (Molecular devices, Sunnyvale, CA, USA), and NO concentration was determined based on the standard curve with sodium nitrite.

### 2.7. Western Blotting

Protein expression in RAW 264.7 cells was quantified by Western blotting, with modification [18]. RAW 264.7 cells were plated in 60-mm culture dishes (8 × 10^5^ cells per well) and incubated overnight at 37 °C and 5% CO_2_. Plated cells were treated with 100 ng/mL of LPS and samples for 20 h. The cells were washed with phosphate buffered saline (PBS), harvested with a radioimmunoprecipitation assay lysis buffer (10 mM Tris-HCl, 5 mM EDTA, 150 mM NaCl, 1% Triton X-100, 1% sodium deoxcholate, 0.1% sodium dodecyl sulfate (SDS), 0.1 mM Na_3_VO_4_, 1% phenylmethylsulfonyl fluoride, 1% protease inhibitor cocktail) to extract cellular proteins. After centrifugation at 4 °C for 15 min (13,000× *g*), 40 μg of cellular protein were separated by 8% SDS-PAGE and transferred to a polyvinylidene difluoride membrane (Millipore, Billerica, MA, USA). After blocking for 1 h, the membrane was incubated with primary antibodies. After incubating with secondary antibodies, the expression levels of proteins were detected by ECL (ATTO, Tokyo, Japan) using EZ capture MG (ATTO) and quantified with CS Analyzer (ver. 3.0, ATTO, Tokyo, Japan).

### 2.8. LC-MS/MS Analysis

Samples were analyzed using an Accela HPLC system with a PDA detector (Accela 80 Hz PDA detector, San Jose, CA, USA) and an LTQ-Velos ion trap-MS fitted with a heat electrospray ionization interface (Thermo Fisher Scientific, San Jose, CA, USA). Separation was carried out using an YMC-Triart C18 column (1.9 µm, 2.0 × 100 mm, YMC Co., Kyoto, Japan) at 36 °C. The flow rate was 0.3 mL/min, with the solvents composed of 0.1% formic acid in water (A) and 0.1% formic acid in acetonitrile (B). The linear gradient profile was as follows: 0–30% of solvent B over 30 min, 30–100% of solvent B over 10 min, then returned to the initial conditions (0% of solvent B) over 5 min and conditioned at 0% of solvent B for 5 min; the run was concluded at 50 min. UV spectrum was measured between 190 and 800 nm. Analysis was carried out using full scan, MS2 and MS3 scanning from *m*/*z* 50–1500 Da. The capillary temperature was 275 °C, and the source voltage was set to 5 kV for the negative and positive ionization modes.

### 2.9. Data Processing and Multivariate Statistical Analyses

The acquired LC-MS/MS data were processed by noise or baseline filtering, feature detection, alignment, and normalization using SIEVE software (Thermo Fisher Scientific, Waltham, MA, USA) with a mass range of 100–1000 Da, retention time of 0.5–30.0 min, *m*/*z* width of 2000 ppm, retention time width of 2.5 min, alignment minimum intensity of 1000, and correlation binary width 2. Multivariate statistical analysis was performed on the acquired data using SIMCA (Umetrics, Umea, Sweden). Principal component analysis (PCA) was used to visualize the similarities and differences between groups of samples, and the S-plot generated by orthogonal partial least-squared discriminant analysis (OPLS-DA) was used to achieve the maximum discrimination and detect significant metabolites in the samples. Both PCA and OPLS-DA were conducted after scaling based on Pareto to reduce the relative importance of large values.

### 2.10. Metabolite Identification

The data acquired from data processing and statistical analyses were used for informative non-targeted metabolite profiling with LC-MS/MS spectra and MS/MS fragmentation patterns. The metabolites were identified after comparison with those proposed by the Metlin database (http://metlin.scripps.edu), Massbank database (www.massbank.jp), and related reports.

## 3. Results 

### 3.1. Preparing Ethanol Extracts of *A. arguta* Leaves

For the partial purification of active compounds, *A. arguta* leaves were extracted using different concentrations of ethanol, and the extraction yields were compared. The 75% ethanol extract exhibited the highest extraction yield (20.15 ± 0.85%), whereas the yields were 10.78 ± 2.15%, 13.68 ± 1.09%, 15.89 ± 1.18%, and 4.71 ± 0.15% after extraction with distilled water, 25% ethanol, 50% ethanol, 75% ethanol, and 100% ethanol, respectively. Thus, the 75% ethanol extract was selected for further investigation.

### 3.2. Anti-Inflammatory Activity of the Extracts and Its Fractions: NO Production in LPS-Induced RAW 264.7 Cells

**Figure 1:**
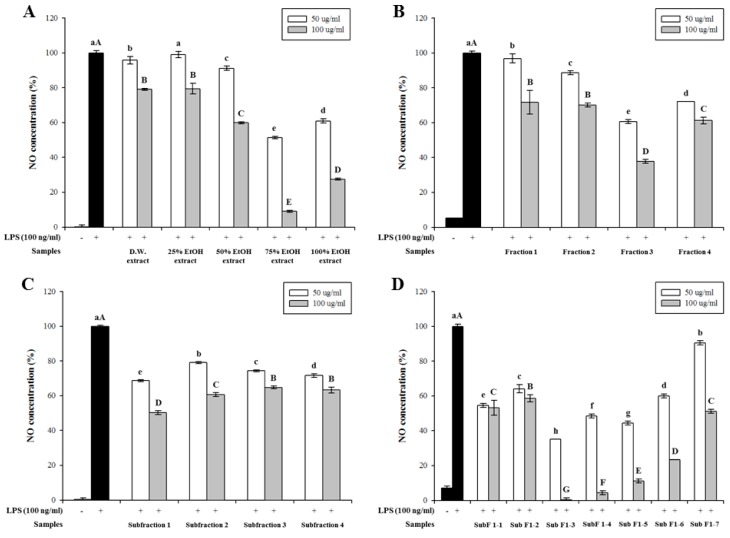
Effect of *Actinidia arguta* (Actinidiaceae) leaves extracts and the fractions obtained from the 75% ethanol extract, prepared using preparative liquid chromatography (prep-LC) on nitric oxide (NO) production in lipopolysaccharide (LPS)-induced RAW 264.7 cell. Cells were treated with 100 ng/mL of LPS and samples (50 and 100 μg/mL) for 20 h, and then NO concentration was measured. Values are expressed as a percentage of control (LPS alone). Data are expressed as mean ± SD after triplicate analysis. Different letters indicate significant differences compared with LPS control at *p* < 0.05. (**A**) *A. arguta* extracts. (**B**) Fractions from the 75% ethanol extract of *A. arguta* leaves. (**C**) Sub-fractions from fraction 3. (**D**) Fractions from sub-fraction 1.

All extracts and their fractions inhibited LPS-induced NO production in a dose-dependent manner. Notably, the 75% ethanol extract exhibited the strongest inhibitory activity at concentrations of 50 (48.7%) and 100 μg/mL (90.0%) (Figure 1A). Considering extraction yield, 75% ethanol was selected as the most effective solvent for extracting anti-inflammatory metabolites from the leaves of *A. arguta*.

First, the 75% ethanol extracts were partially purified by prep-LC. Fraction 3 showed the highest inhibitory effect on NO production at concentrations of 50 (39.4%) and 100 μg/mL (62.3%) (Figure 1B). Thus, active fraction 3 was further purified by prep-LC. Sub-fraction 1 had a strong inhibitory effect on NO production compared to that of other sub-fractions at concentrations of 50 (31.4%) and 100 μg/mL (49.8%) (Figure 1C). Finally, seven sub-fractions (sub-fractions 1-1 to 1-7) were obtained and their activities were evaluated (Figure 1D). Sub-fraction 1-3 showed the strongest inhibitory effect on NO production at concentrations of 50 (64.8%) and 100 μg/mL (99.7%) in a dose-dependent manner. Cytotoxicities of the extracts and their fractions were measured using the MTT assay, and no cytotoxicity was observed on RAW 264.7 cells at these concentrations (50 and 100 μg/mL).

### 3.3. Inhibitory Effect on iNOS Expression in LPS-Stimulated RAW 264.7 Cells

**Figure 2:**
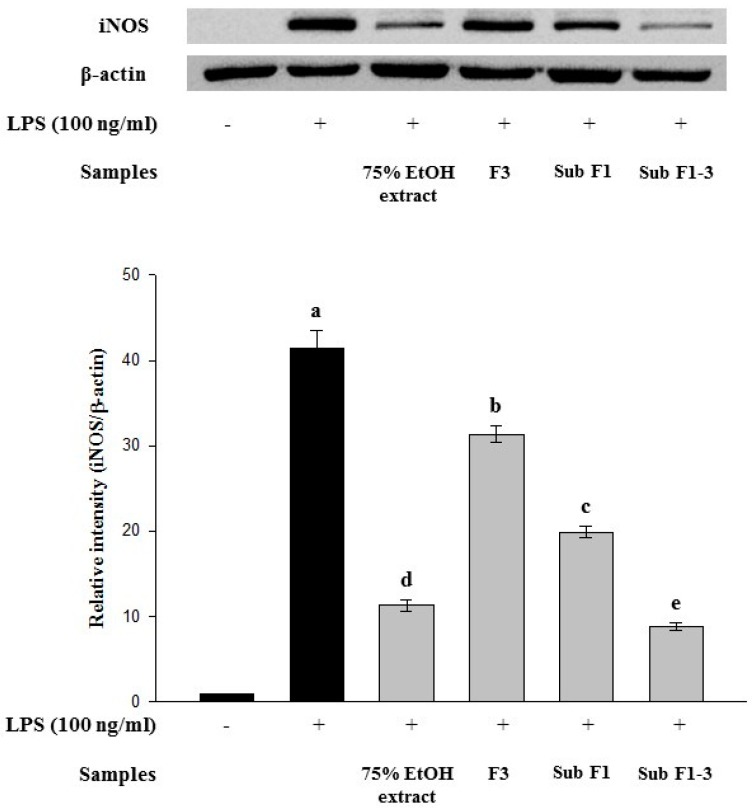
Effect of *Actinidia arguta* leaves extracts and its partially purified fractions on inducible nitric oxide synthase (iNOS) expression in lipopolysaccharide (LPS)-induced RAW 264.7 cells. Cells were treated with LPS (100 ng/mL) as well as the extracts and their partially purified fractions (50 μg/mL) for 20 h. Cell lysates were prepared and subjected to Western blotting using antibodies specific for iNOS and β-actin. Data are expressed as mean ± SD after triplicate analysis. Different letters indicate significant differences compared with LPS control at *p* < 0.05. (F3: fraction 3, Sub F1: sub-fraction 1, and Sub F1-3: sub-fraction 1-3).

LPS increased iNOS expression (Figure 2); however, the 75% ethanol extract, fraction 3, sub-fraction 1, and sub-fraction 1-3 suppressed LPS-induced iNOS expression. Furthermore, sub-fraction 1-3, the final partially purified fraction, showed the highest inhibitory effect on LPS-induced iNOS expression.

### 3.4. Multivariate Statistical Analyses

**Figure 3:**
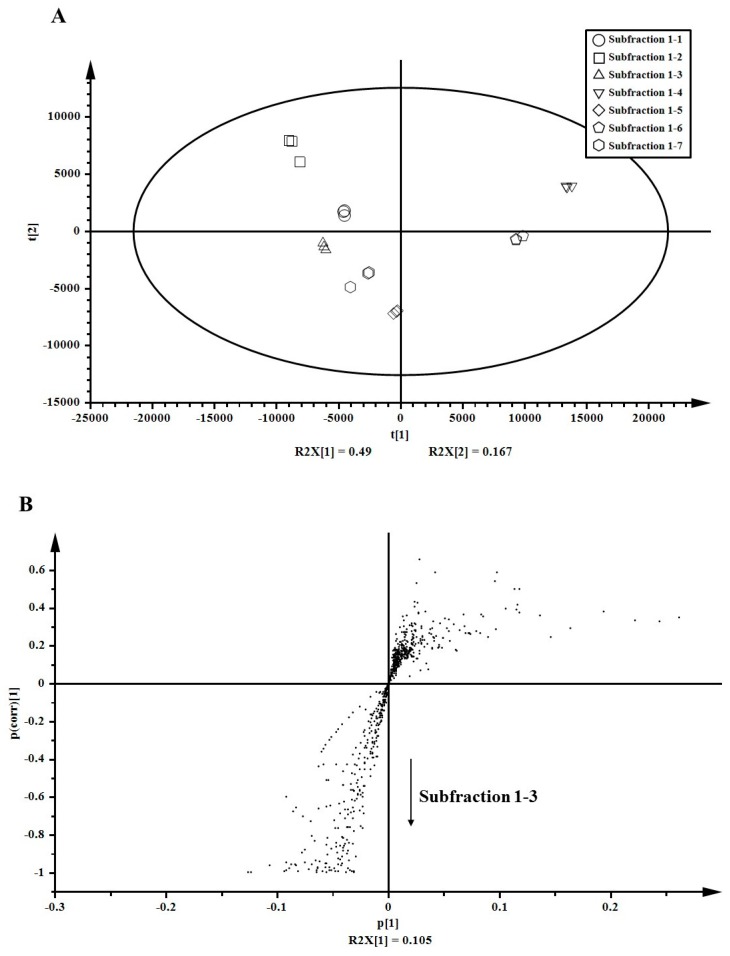
Multivariate statistical analyses for metabolites in the sub-subfractions obtained from the 75% ethanol extract of *Actinidia arguta* leaves. (**A**) Score plot generated by principal component analysis (PCA). (**B**) S-plot generated by orthogonal partial least-squared discriminant analysis (OPLS-DA).

After partial purification using prep-LC, sub-fraction 1-3 was finally purified from the 75% ethanol extract of *A. arguta* leaves. To identify key anti-inflammatory compounds, metabolites in sub-fractions (sub-fractions 1-1 to 1-7) were analyzed using LC-ESI-ion trap-MS/MS. Normalized 654 peaks were conclusively obtained after raw data processing by SIEVE and were subjected to multivariate statistical analyses (PCA and OPLS-DA) for estimating any statistically significant differences among sub-fractions 1-1 to 1-7. PCA score plots demonstrated distinctive metabolite phenotypes of the sub-fractions. PC1 (t1) explained 49.0% of the variation and PC2 (t2) accounted for 16.7% variation. Sub-fraction 1-3 positively correlated with sub-fractions 1-5 and 1-7, and negatively correlated with sub-fraction 1-4 (Figure 3A). The S-plot derived from OPLS-DA revealed variables that changed the most in an uncoordinated way were at the top or bottom of the plot. The variables for sub-fraction 1-3 were located at the bottom, and those for the rest of the sub-fractions were located at the top (Figure 3B). At the bottom of the S-plot, 33 variables were significantly discriminated (*p* < 0.05). Based on these results, variables for sub-fraction 1-3 were identified to confirm the correlation between identified metabolites and their anti-inflammatory effects.

### 3.5. Metabolite Identification

**Figure 4:**
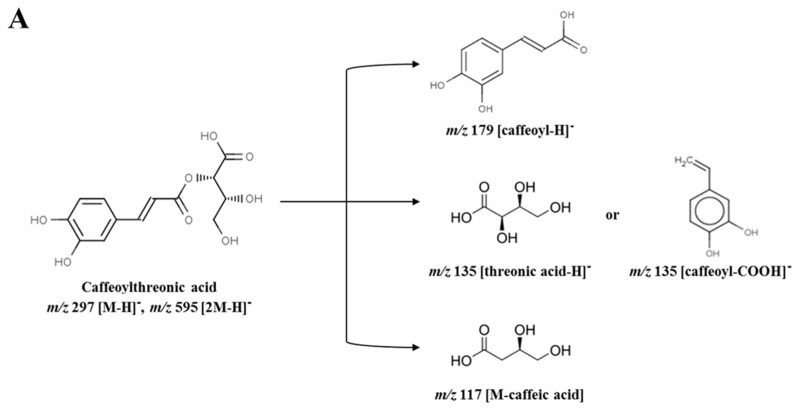

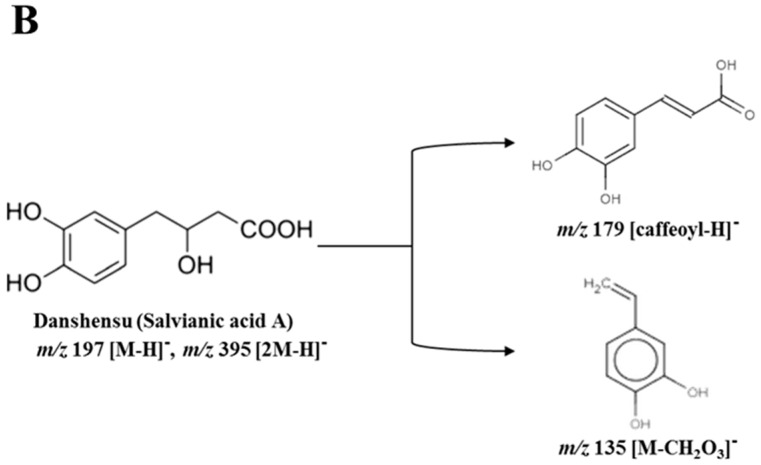
Fragmentary patterns of (**A**) caffeoylthreonic acid and (**B**) danshensu in sub-fraction 1-3 obtained from the 75% ethanol extract of *Actinidia arguta* leaves.

Among 33 variables, 4 metabolites were tentatively identified as putative anti-inflammatory compounds in sub-fraction 1-3. The identified metabolites were caffeoylthreonic acid dimer (RT (retention time) 14.79 min, *m*/*z* 595.08), danshensu (salvianic acid A; RT 7.56 min, *m*/*z* 395.08), caffeic acid derivative (RT 16.10 min, *m*/*z* 309.08), and caffeoylthreonic acid (RT 14.74 min, *m*/*z* 297.06) (Table 1). Caffeoylthreonic acid and its dimer were located at −0.094 (PC1) and −0.990 (P(corr) [1]) and −0.124 (PC1) and −0.993 (P(corr) [1]), respectively. Danshensu was also located at −0.056 (PC1) and −0.992 (P(corr) [1]) (Figure 3B). These results indicate that these compounds are the major distinctive metabolites in sub-fraction 1-3. Caffeoylthreonic acid showed a molecular ion of *m*/*z* 297.06 [M − H]^−^, indicating the molecular formula C_13_H_14_O_8_. The fragment ion of the molecular ion *m*/*z* 297.06 yielded *m*/*z* 178.99 [caffeoyl − H]^−^, *m*/*z* 134.97 [threonic acid − H]^−^ or [caffeoyl − COOH]^−^, and *m*/*z* 117.03 [M − caffeic acid]. Caffeoylthreonic acid dimer (*m*/*z* 595.08, [2M − H]^−^) also yielded *m*/*z* 297.04 [M − H]^−^, *m*/*z* 178.97 [caffeoyl − H]^−^, *m*/*z* 134.97 [threonic acid − H]^−^, and *m*/*z* 116.96 [M − caffeic acid]^−^ (Parveen, et al. [19], Figure 4A). Danshensu showed a molecular ion of *m*/*z* 395.08 [2M − H]^−^, indicating the molecular formula C_9_H_10_O_5_. The fragment ion of the molecular ion *m*/*z* 395.08 yielded *m*/*z* 197.03 [M − H]^−^, *m*/*z* 179.05 [caffeoyl − H]^−^, and *m*/*z* 135.02 [M − CH_2_O_3_]^−^ (Chen, et al. [20], Figure 4B).

## 4. Discussion

The leaves of *A. arguta* are used as a traditional medicine in Korea due to their anti-diabetic, antioxidant, and anti-inflammatory effects [8,9]. However, there are some reports on the systematic analysis of the bioactive components in the leaves. In the present study the anti-inflammatory effects of *A. arguta* leaves were investigated, and anti-inflammatory compounds in *A. arguta* leaves were screened and identified through metabolomic analysis. Moreover, anti-inflammatory compounds in *A. arguta* leaves were screened and identified through metabolomic analysis. The leaves of *A. arguta* were extracted with ethanol at different concentrations and partially purified using in vitro bioassay-guided fractionation to screen potent anti-inflammatory metabolites. To confirm the anti-inflammatory effects, RAW 264.7 cells were treated with the extracts and their fractions along with LPS (100 ng/mL). Because the 75% ethanol extract exhibited the highest extraction yield and had the strongest inhibitory effect on LPS-induced NO production, 75% ethanol was selected as the most effective extraction solvent. Through partial purification, sub-fraction 1-3 was finally purified from the *A. arguta* leaves extract. Sub-fraction 1-3 inhibited iNOS expression better than the 75% ethanol extract, fraction 3, and sub-fraction 1; therefore, major active compounds in this sub-fraction were analyzed by LC-MS/MS with multivariate statistical analyses (PCA and OPLS-DA). Significant variation of metabolite profiles in each fraction were clearly characterized by PCA analysis, and the following OPLS-DA analysis helped to screen out critical contributors for the variation. Similar demonstrations on metabolomic screening of active compounds from foods were reported, based on non-targeted profiling and screening [8,17,18]. These approaches could reveal a new compound while the classical methods only identify and compare selected groups of compounds [21,22].

Caffeoylthreonic acid and danshensu (salvianic acid A) were identified as major potent anti-inflammatory compounds of *A. arguta*. Caffeoylthreonic acid is an ester of caffeic acid and threonic acid. Danshensu is also called salvianic acid A, or 3,4-dihydroxyphenyl lactic acid; it is a caffeic acid derivative that is present in many plants as a secondary metabolite [23]. Danshensu has been reported to exhibit antioxidant [24] and anti-inflammatory activities [25,26]. The anti-inflammatory activities of caffeoylthreonic acid and danshensu are reported to be correlated with their chemical structures, which resemble that of caffeic acid. Caffeic acid has been extensively studied for its antioxidant and anti-inflammatory effects [27,28,29]. These effects are expressed not only by caffeic acid, but also its derivatives. For instance, caffeic acid phenethyl ester inhibits NO production by inhibiting iNOS gene transcription via its action on NF-κB sites in the iNOS promoter, as well as by directly inhibiting the catalytic activity of iNOS [30]. In addition, caffeic acid derivatives exert in vitro and in vivo anti-inflammatory actions by scavenging NO and modulating iNOS expression [31]. Thus, caffeoylthreonic acid and danshensu could be expected to exert anti-inflammatory activities similar to those of caffeic acid derivatives.

This is the first study to demonstrate the anti-inflammatory effect of caffeic acid derivatives isolated from the extract of *A*. *arguta* leaves; these derivatives were identified by metabolomic analysis to be caffeoylthreonic acid and danshensu, the key anti-inflammatory compounds.

## 5. Conclusions

The results demonstrated that metabolomic analysis could be a useful tool for screening anti-inflammatory compounds in medicinal plant materials without an extensive separation and purification process. An anti-inflammatory effect was observed in ethanol extracts of *A. arguta* leaves through iNOS suppression, and the key metabolites were explored through metabolomic analysis. Simple multivariate analysis on the metabolites successfully showed up caffeic acid derivatives as candidates, then caffeoylthreonic acid and danshensu were identified as the key anti-inflammatory compounds.

## Figures and Tables

**Table 1:** Tentative identification of the key anti-inflammatory compounds in sub-fraction 1-3 from the 75% ethanol extract of *Actinidia arguta* leaves.

*m*/*z* [M − H]^−^	UV λ_max_ (nm)	MS2 (MS3)	Tentative Identification	References
595.08	218, 303, 324	297.04 (178.97, 134.97, 116.96)	Caffeoylthreonic acid dimer	Parveen et al. (2008)
395.08	224, 286	197.03 (179.02, 153.05, 135.03, 73.03), 179.05, 153.11, 135.02	Danshensu (Salvianic acid A) dimer	Chen et al. (2011)
309.08	219, 285, 319	179.00 (134.95), 161.02, 135.03	Caffeic acid derivative	Metlin
297.06	219, 298, 324	178.99, 134.97, 117.03	Caffeoylthreonic acid	Parveen et al. (2008)

MS2, first generation of product ion spectra; MS3, second generation product ion spectra.

## Abbreviations

MTT, 3-(4,5-dimethylthiazol-2-yl)-2,5-diphenyltetrazolium bromide; DMEM, dulbecco’s modified eagle’s medium; DPBS, dulbecco’s phosphate buffered saline; FBS, fetal bovine serum; iNOS, inducible nitric oxide synthase; LPS, lipopolysaccharide; NO, nitric oxide; NOS, nitric oxide synthase; OPLS-DA, orthogonal partial least-squared discriminant analysis; Prep-LC, preparative LC; PCA, principal component analysis.
